# A nomogram for predicting risk factors of testicular salvage after testicular torsion in children

**DOI:** 10.1111/iju.15420

**Published:** 2024-02-10

**Authors:** Pengyu Chen, Weipeng Huang, Yingying He, Mengkui Sun, Xuerui Sun, Yiyan Huang, Shoulin Li

**Affiliations:** ^1^ Department of Urology Shenzhen Children's Hospital Shenzhen Guangdong People's Republic of China; ^2^ Department of Critical Care Medicine Sir Run Run Shaw Hospital, Zhejiang University School of Medicine Hangzhou People's Republic of China; ^3^ Department of Nursing Sir Run Run Shaw Hospital, Zhejiang University School of Medicine Hangzhou People's Republic of China

**Keywords:** hemodynamics, monocytes, orchiectomy, orchiopexy, testicular torsion

## Abstract

**Objectives:**

This study aimed to establish a nomogram for predicting the probability of testicular salvage after testicular torsion in children.

**Methods:**

We retrospectively collected data of children with testicular torsion who were treated at Shenzhen Children's Hospital between September 2005 and August 2022. Of the training cohort, 113 patients who underwent orchiectomy and five with testicular atrophy after orchiopexy were included in the failed testicular salvage group. Additionally, 37 patients who underwent orchiopexy without postoperative testicular atrophy were included in the successful testicular salvage group. The predictive factors affecting testicular salvage were determined using univariate and multivariate logistic regression analyses; a nomogram was constructed. The nomogram was verified using data from the validation group.

**Results:**

Using multivariate logistic regression analysis, the independent risk factors of testicular salvage after testicular torsion were symptom duration (*p* = 0.034), intratesticular blood flow (*p* = 0.003), spermatic cord torsion degree (*p* = 0.037), and monocyte count (odds ratio: 0.012, *p* = 0.036). A nomogram was established based on these four risk factors. In the training cohort, the area under the receiver operating characteristic curve was 0.969. The area under the receiver operating characteristic curve of the verification cohort was 0.965, indicating good discrimination ability of the nomogram. Increased symptom duration without intratesticular blood flow increased the monocyte count and spermatic cord torsion degree and decreased the success rate of testicular salvage.

**Conclusion:**

This prediction model could obtain the corresponding probability of testicular salvage according to the clinical characteristics of different patients with testicular torsion, providing reference for clinicians and parents.

**Abbreviations & Acronyms**
AUC: area under the curve
CI: confidence interval
MPV: mean platelet volume
NLR: neutrophil‐lymphocyte ratio
OR: odds ratio
PLR: platelet‐lymphocyte ratio
ROC: receiver operating characteristic

## INTRODUCTION

Testicular torsion is a common emergency in pediatric urology. The incidence of testicular torsion is approximately 0.004% in patients under 18 years of age, accounting for 52.5% of scrotal emergencies in children.^1^, ^2^ Testicular torsion is the rotation of the testis and epididymis around the spermatic vessels. This causes testicular blood flow to either decrease or stop entirely. If the rotated state is not corrected in time, it could easily cause testicular ischemic damage and even necrosis.^3^ Some studies have pointed out that the success rate of testicular rescue within 6 h after testicular torsion is 90%–100% and drops to 0%–10% after 12–24 h.^4^ In patients with testicular torsion, surgical exploration must be performed as soon as possible to restore the normal anatomical state of the testis and spermatic cord. Moreover, orchiectomy or orchiopexy should be considered based on testicular vitality. Of note, orchiopexy did not indicate successful testicular salvage. Testicular atrophy occurs in 16%–48% of patients who undergo orchiopexy after testicular torsion.^5^, ^6^ In this study, the process of diagnosis and treatment of testicular torsion was regarded as a whole, and the relatively objective indices in patients with testicular torsion from onset to long‐term follow‐up were extracted and included in the study; the effects of various factors on testicular salvage were analyzed. We also established a prediction model for testicular salvage, which could provide a reference for clinicians and parents to understand the probability of testicular salvage in children with testicular torsion.

## METHODS

### Patients' characteristics

This study was approved by the Institutional Review Board of Shenzhen Children's Hospital (No. 2022014). Using the medical record system of Shenzhen Children's Hospital, the medical records of children with testicular torsion treated in our hospital from September 2005 to August 2022 were extracted and reviewed. The dataset of this study consisted of two parts: training and verification cohorts. Children from September 2005 to January 2021 were included in the training cohort; a prediction model for testicular rescue was established based on the data of this cohort. We used children diagnosed with testicular torsion from February 2021 to August 2022 as the verification cohort for the predictive model to test the accuracy of the nomogram.

The inclusion criteria were the following: First, the patient's age being <18 years. Second, the children have been examined by a urologist at our hospital before surgery and underwent ultrasound examinations by ultrasound specialists at our hospital. Third, testicular torsion has been surgically confirmed. Fourth, the patients have been followed up for a postoperative period of at least 6 months. The exclusion criteria were the following: presence of a neonatal testicular torsion and presence of incomplete medical records or follow‐up data.

In this study, the patients' clinical data were collected, including age; side of testicular torsion; clinical symptoms and signs; duration of symptoms (the time between the occurrence of symptoms and the beginning of surgery); ultrasound results; operation‐related data; hematological parameters; and postoperative follow‐up data.

Ultrasonographic examination was performed using Vivid 7, Voluson E8, and Logiq E9 color ultrasound instruments (GE Healthcare, Chicago, USA). With patients in the supine position, bilateral retroperitoneal, inguinal, scrotal, and testicular ultrasonography was performed. The position, shape, size, boundary, echo, color Doppler blood flow signal, and periphery of the testis were detected. Testicular volume was estimated using the ellipsoid formula (V=L∙W∙H∙0.523).

### Surgical procedures

After admission, all patients with scrotal emergency underwent physical examination, ultrasound, and blood routine examination. When testicular torsion was suspected, rapid treatment channels were provided immediately to ensure surgical exploration within 1 h. The children were placed in the supine position. After adequate anesthesia, according to the position of the testes, a groin incision or transverse scrotal incision was made to explore the spermatic vessels. If torsion of the spermatic vessels was found, the testis was raised out of the thecal capsule and restored. Blood circulation in the affected testis was observed after heating with warm water for 20 min. According to the Arda grade, to decide whether to retain the testis, we cut open the white membrane of the testis and observed bleeding in the section. The grades were Arda grade I, immediate blood oozing; Arda grade II, fresh blood exudation within 10 min; and Arda grade III, no fresh blood exudation beyond 10 min. Testicular preservation and orchiopexy were performed in those with Arda grades I and II, whereas orchiectomy was considered for grade III.^7^

### Grouping design

According to previous literature, testicular atrophy was defined as a volume difference of ≥50% between the affected and healthy testes after orchiopexy.^8^, ^9^, ^10^ The training cohort was divided into two groups: the successful and failed testicular salvage groups. The patients who received orchiectomy and those who experienced testicular atrophy following orchiopexy were categorized into the failed testicular salvage group, whereas those who did not develop testicular atrophy during follow‐up were categorized into the testicular rescue success group.^2^

### Statistical analysis

This study primarily used SPSS 22.0 (IBM, Armonk, NY, USA), R 3.6.0 (R Foundation for Statistical Computing, Vienna, Austria), and MedCalc 20.0.8 (Ostend, Belgium) for data analysis. First, a univariate analysis was performed to determine the relationship between all variables and testicular rescue after testicular torsion. To identify independent risk factors for testicular rescue, multivariate logistic regression analysis was performed. All significant predictors were analyzed using reduced‐model multivariate analysis; the results were used to establish the prediction model formula. Finally, based on the prediction model formula, the nomogram was constructed using the rms package of R software. Finally, the bootstrap method was used to verify the nomogram by repeatedly sampling the verification group data for 1000 times. The receiver operating characteristic (ROC) curve of the participants was established. The area under the curve (AUC) was evaluated; the sensitivity and specificity were calculated. Categorical variables are presented as number of cases and percentages. Non‐normally distributed continuous variables are presented as median with interquartile range.

## RESULTS

Between September 2005 and January 2021, 187 children presented to our hospital with testicular torsion. Twenty cases of neonatal onset, seven of loss to follow‐up after surgery, four missing the results of preoperative ultrasound examination in our hospital, and one patient whose parents refused surgical treatment in our hospital were excluded from the study. Ultimately, 155 patients with testicular torsion were included in the training cohort. The median age of the training cohort was 8.98 years (2.75–12.53 years): 11.31 years (9.35–12.77 years) in the successful testicular rescue group and 6.91 years (1.59–12.48 years) in the testicular salvage failure group. A total of 113 patients underwent orchiectomy and 42 patients underwent orchiopexy, of which five developed testicular atrophy and were diagnosed with testicular salvage failure (Table 1).

**TABLE 1:** Baseline characteristics of patients with testicular torsion in the training cohort.

Variable	Successful salvage	Failed salvage	*p*
Total	37	118	
Age (year)	11.31 (9.35, 12.77)	6.91 (1.59, 12.48)	0.001
Affected side			0.042
Left	23	93	
Right	14	25	
Clinical characteristics			
Duration of symptoms (hour)	6 (4, 24)	48 (24, 72)	<0.001
Vomiting	2	17	0.242
Cryptorchid	3	19	0.224
Ultrasound results			
Testicular echotexture			<0.001
Homogeneous	15	2	
Nonhomogeneous	22	116	
Intratesticular blood flow			<0.001
Visible	23	4	
Invisible	14	114	
Intraoperative condition			
Orchidopexy	37	5	
Orchiectomy	0	113	
Torsion angle (°)	360 (180, 540)	540(360, 720)	<0.001
Hematological parameters			
WBC (10^9^/L)	9.67 (7.47, 13.47)	11.9(9.97, 14.04)	0.006
Neutrophil (10^9^/L)	6.58 (4.37, 9.69)	7.06 (4.99, 9.90)	0.511
Lymphocyte (10^9^/L)	1.96 (1.36, 3.43)	2.87 (1.95, 5.40)	0.002
Monocyte (10^9^/L)	0.40 (0.30, 0.64)	0.68 (0.54, 0.92)	<0.001
Eosinophil (10^9^/L)	0.10 (0.02, 0.21)	0.13 (0.05, 0.35)	0.095
Basophil (10^9^/L)	0.03 (0.02, 0.05)	0.04 (0.02, 0.06)	0.244
MPV (fl)	9.70 (8.65, 10.25)	9.70 (9.10, 10.40)	0.326
Platelet (10^9^/L)	309.0 (270.00356.50)	320.5 (271.50, 399.75)	0.326
NLR	3.32 (1.41, 7.96)	2.11 (1.07, 4.86)	0.046
PLR	144.62 (97.82, 216.26)	112.26 (66.07, 148.27)	0.001

Abbreviations: MPV, mean platelet volume; NLR, neutrophil‐lymphocyte ratio; PLR, platelet‐lymphocyte ratio; WBC, white blood cell.

Univariate logistic regression analysis showed that the success of testicular salvage after testicular torsion could be related to the following factors (Table 2): age (*p* = 0.001), affected side (*p* = 0.045), duration of symptoms (*p* < 0.001), testicular parenchymal echotexture (*p* < 0.001), intratesticular blood flow (*p* < 0.001), spermatic cord torsion degree (*p* < 0.001), white blood cell count (*p* = 0.008), monocyte count (*p* < 0.001), neutrophil‐lymphocyte ratio (NLR) (*p* = 0.022), and platelet‐lymphocyte ratio (PLR) (*p* = 0.001).

**TABLE 2:** Univariate and multivariate analysis results.

Variable	Univariate analysis		Multivariate analysis	
	*p*	OR (95%CI)	*p*	OR (95% CI)
Age	0.001	1.171 (1.071–1.280)	0.247	1.175 (0.894–1.545)
Affected side	0.045	0.442 (0.199–0.981)	0.791	1.280 (0.205–7.990)
Symptom duration	<0.001	0.961 (0.942–0.980)	0.034	0.970 (0.943–0.998)
Cryptorchid	0.234	0.460 (0.128–1.651)		
Vomiting	0.162	2.946 (0.648–13.397)		
Testicular echotexture	<0.001	0.025 (0.005–0.118)	0.075	0.011 (0.000–1.569)
Intratesticular blood flow	<0.001	0.021 (0.006–0.071)	<0.001	0.003 (0.000–0.070)
Torsion angle	<0.001	0.994 (0.992–0.997)	0.037	0.995 (0.991–1.000)
WBC	0.013	0.856 (0.757–0.967)	0.255	0.714 (0.401–1.274)
Monocyte	<0.001	0.012 (0.002–0.086)	0.036	0.012 (0.000–0.755)
Eosinophil	0.279	0.377 (0.065–2.202)		
Basophil	0.196	0.000 (0.000–425.896)		
MPV	0.863	0.969 (0.674–1.392)		
PLT	0.279	0.998 (0.994–1.002)		
NLR	0.022	1.128 (1.018–1.250)	0.634	1.140 (0.664–1.960)
PLR	0.001	1.007 (1.003–1.012)	0.942	1.001 (0.985–1.016)

Abbreviations: CI, confidence interval; MPV, mean platelet volume; NLR, neutrophil‐lymphocyte ratio; OR, odds ratio; PLR, platelet‐lymphocyte ratio; WBC, white blood cell.

**FIGURE 1:**
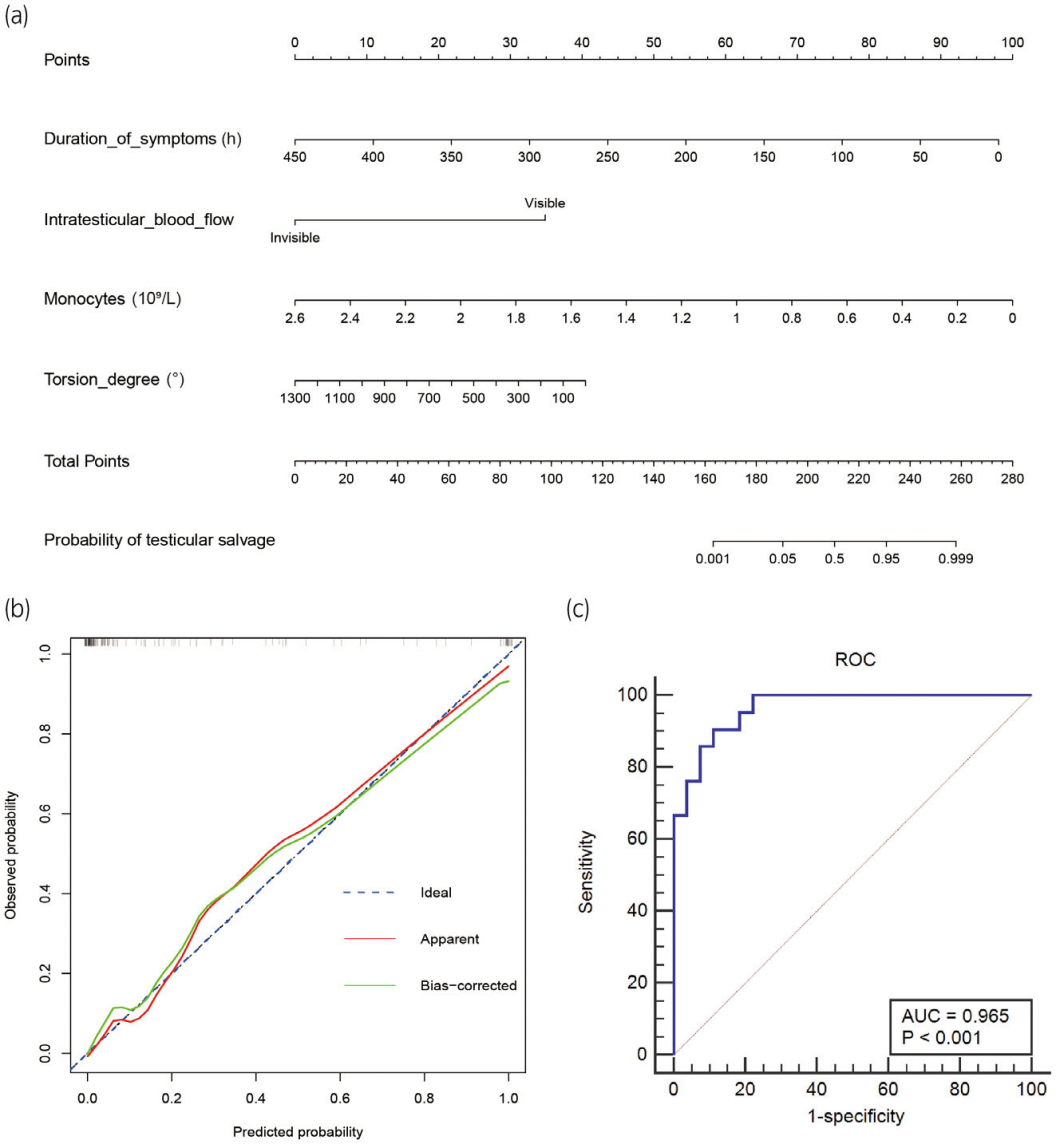
(a) Nomogram for the prediction of testicular salvage after testicular torsion in children. Prognostic nomograms including the duration of symptoms, intratesticular blood flow, monocyte count, and spermatic cord torsion degree. (b) Calibration plot. (c) Receiver operating characteristic (ROC) curve of the verification cohort.

**FIGURE 2:**
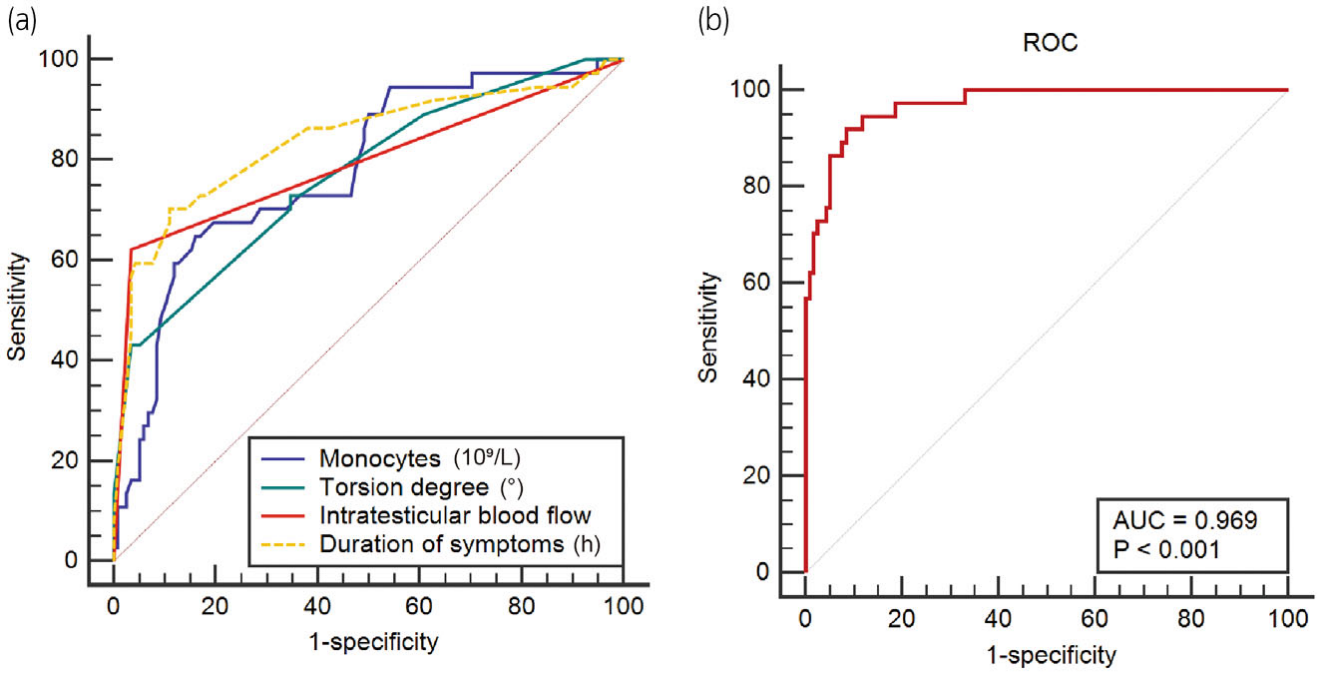
(a) Receiver operating characteristic (ROC) curves of the duration of symptoms, intratesticular blood flow, monocyte count, and spermatic cord torsion degree for predicting testicular salvage. (b) ROC curve of the training cohort.

Multivariate logistic regression analysis was used to analyze statistically significant factors from the univariate analysis (Table 2). The predictors of successful testicular rescue after testicular torsion were the duration of symptoms (odds ratio [OR], 0.970; 95% confidence interval [CI], 0.943–0.998; *p* = 0.034), intratesticular blood flow (OR, 0.003; 95% CI, 0.000–0.070; *p* < 0.001), spermatic cord torsion degree (OR, 0.995; 95% CI, 0.991–1.000; *p* = 0.037), and monocyte counts (OR, 0.012; 95% CI, 0.000–0.755; *p* = 0.036). When the duration of symptoms after testicular torsion was long, there was no intratesticular blood flow, the degree of spermatic cord torsion was large, the monocyte count increased, and the success rate of testicular rescue decreased. We constructed a nomogram based on these independent risk factors (Figure 1a). The performance of the constructed nomogram was evaluated using a calibration plot (Figure 1b). ROC analysis of the predictive factors showed that the AUC of monocytes was 0.781 (sensitivity, 83.9%; specificity, 64.9%; cut‐off, 0.485 × 109/L). The AUC of spermatic cord torsion degree was 0.770 (sensitivity, 96.6%; specificity, 43.2%; cut‐off, 225°). The AUC of symptom duration was 0.838 (sensitivity, 89.0%; specificity, 70.3%; cut‐off, 14.5 h) (Figure 2a). The AUC of testicular blood flow was 0.794. The AUC of the ROC curve of the training cohort was 0.969 (sensitivity, 91.9%; specificity, 92.4%; cut‐off, 0.268) (Figure 2b).

From February 2021 to August 2022, 48 patients with testicular torsion were enrolled in the validation cohort (Table 3). The clinical records of the 48 patients in the verification cohort were fitted into the predictive model for verification. The results showed that the area under the ROC curve was 0.965 (95% CI, 0.867–0.997). When the cut‐off value was 0.431, the sensitivity and specificity of the prediction model were 90.5% and 88.9%, respectively (Figure 1c).

**TABLE 3:** Baseline characteristics of patients with testicular torsion in the verification cohort.

Variable	Successful salvage	Failed salvage	*p*
Total	21	27	
Age (year)	11.45 (4.46, 13.02)	11.65 (7.15, 13.92)	0.366
Affected side			0.883
Left	12	16	
Right	9	11	
Clinical characteristics			
Duration of symptoms (hour)	6 (2.5, 12)	24 (20, 72)	<0.001
Vomiting	4	2	
Cryptorchid	2	5	
Ultrasound results			
Testicular echotexture			<0.001
Homogeneous	11	0	
Nonhomogeneous	10	27	
Intratesticular blood flow			<0.001
Visible	12	2	
Invisible	9	25	
Intraoperative condition			
Orchidopexy	21	5	
Orchiectomy	0	22	
Torsion angle (°)	360 (180, 450)	540 (360, 540)	0.066
Hematological parameters			
WBC (10^9^/L)	10.90 (8.05, 13.21)	12.6 (9.86, 15.12)	0.086
Neutrophil (10^9^/L)	7.63 (5.18, 10.34)	8.04 (6.17, 11.05)	0.324
Lymphocyte (10^9^/L)	2.08 (1.41, 3.01)	2.69 (1.80, 3.43)	0.140
Monocyte (10^9^/L)	0.44 (0.32, 0.58)	0.79 (0.59, 0.94)	<0.001
Eosinophil (10^9^/L)	0.11 (0.04, 0.22)	0.09 (0.03, 0.18)	0.459
Basophil (10^9^/L)	0.03 (0.02, 0.05)	0.04 (0.02, 0.06)	0.230
MPV (fl)	9.60 (9.05, 10.45)	9.00 (9.50, 10.20)	0.560
Platelet (10^9^/L)	286.0 (225.5, 342.0)	311.0 (293.0, 349.0)	0.103
NLR	3.52 (1.92, 7.26)	3.59 (2.19, 5.89)	0.779
PLR	623.70 (506.9, 845.6)	401.1 (295.9, 584.1)	0.513

Abbreviations: MPV, mean platelet volume; NLR, neutrophil‐lymphocyte ratio; PLR, platelet‐lymphocyte ratio; WBC, white blood cell.

## DISCUSSION

Determining testicular viability after testicular torsion is a major challenge in clinical practice. Currently, there are few studies on prediction models of testicular torsion. Zheng et al. predicted the possibility of testicular salvation based on the duration of symptoms, testicular parenchymal echotexture, and intratesticular blood flow in patients with testicular torsion.^11^ However, this study only focused on whether orchiectomy was performed during surgery, wherein a long‐term follow‐up of testicular atrophy in patients with orchiopexy was not performed. We suggest that considering testicular preservation during surgery does not mean successful testicular salvage of torsion; the process of ischemia‐reperfusion injury should be regarded as a whole. When there is no testicular atrophy after orchiopexy, testicular salvage could be regarded as successful. The AUC of our predictive model was 0.965, indicating good predictability. This prediction model could not only provide a reference for surgeons to judge testicular activity during surgery but also answer parents' questions about long‐term prognosis.

Testicular torsion could occur in all age groups; its onset is bimodal. The first peak occurs during the newborn period, while the second peak takes place in teenagers between the ages of 12 and 14 years.^12^, ^13^ In this study, considering that some of the patients with neonatal testicular torsion had intrauterine torsion, there was inevitably a deviation in the statistics of such children's clinical data; thereby, neonatal testicular torsion was contained in the exclusion criteria. Wang et al. showed that the average age of patients undergoing orchiopexy was 11.00 ± 3.44 years, which was significantly higher than that in patients undergoing orchiectomy (6.76 ± 5.56 years).^14^ In this study, the age of onset in the successful testicular rescue and failed testicular salvage groups was 11.31 and 6.91 years, respectively. Although the difference between the two groups was statistically significant, multivariate logistic regression analysis revealed that age was not an independent risk factor for testicular rescue.

The duration of symptoms refers to the time from the onset of symptoms to surgical correction of torsion. This index could reflect the duration of testicular ischemia to a certain extent and is of great significance for the prediction of testicular rescue. Howe et al. conducted a retrospective analysis of the clinical data of 81 patients with testicular torsion and found that the average duration of symptoms in patients who underwent successful testicular salvage was 6.2 h (1–21 h) and 13.4 h (3–24 h) in those who underwent unsuccessful testicular salvage.^2^

Ultrasonography could reveal the characteristics of the testicular parenchyma and blood perfusion, evaluating the vitality of the testicular parenchyma.^15^ The sensitivity of Doppler ultrasound in testicular torsion was 69%–91%, and the specificity was 87%–100%.^16^ If the ultrasonic texture of the twisted testis is normal, the possibility of testicular salvage could be higher. If some or all testes are hypoechoic, this suggests that there is an infarct in the testes. When secondary bleeding occurs, there may be a partial echo enhancement area around the infarcted area.^17^ Boettcher et al. revealed that the combination of clinical features and ultrasound results may improve the specificity of testicular torsion diagnoses, such as the rapid appearance of clinical symptoms and decrease or loss of central blood perfusion on color Doppler ultrasound, which often require early surgical exploration.^15^ In this study, multivariate logistic regression analysis showed that intratesticular blood flow on ultrasound was an independent risk factor for predicting testicular salvage (OR, 0.003; 95% CI, 0.001–0.070; *p* < 0.001). Ultrasound is highly dependent on operator experience and also necessary for guarding against the occurrence of false‐negative results.^16^, ^18^ In patients with suspected testicular torsion, testicular conditions could be determined using ultrasound. However, surgical exploration is still needed as soon as possible.

The blood supply to the testes mainly comes from the spermatic vessels. Therefore, their degree of torsion could reflect testicular ischemia in patients with testicular torsion to a certain extent. It has been indicated that when the torsion of spermatic cord vessels exceeded 450°, the blood supply to the testes would be completely interrupted.^19^ Howe et al. pointed out that when intraoperative exploration found that the torsion of the spermatic cord was >360°, the risk of testicular irreparability was higher.^2^ Clarifying that the degree of spermatic cord torsion only reflects the blood supply of the affected testis, and the greater the degree of torsion, the shorter the time required to cause testicular vascular injury is needed.^20^ Therefore, it does not mean that the greater the degree of spermatic cord torsion is, the more serious the testicular injury is, whereas the former also needs to be combined with more factors, such as the duration of symptoms.

Hematological parameters may be used to predict testicular viability in individuals with testicular torsion. However, the outcomes are inconsistent. He et al. reported that the symptom duration, degree of spermatic cord torsion, and mean platelet volume (MPV) could all be utilized as indicators of the viability of the twisted testicles.^21^ In line with these findings, NLR could also be utilized as a predictor of testicular viability in patients with testicular torsion within 3–12 h, according to other research.^22^ Yilmaz et al. found that monocyte count was the only significant variable of testicular viability (OR, 0.046; 95% CI, 0.006–0.366; *p* = 0.004) in patients with testicular torsion using multivariate analysis.^23^ The authors noted that the monocyte count in patients with testicular torsion might indicate testicular viability. In our study, monocyte count was identified as an independent risk factor for testicular salvage during testicular torsion using multivariate logistic regression analysis (OR, 0.012; 95% CI, 0.000–0.755; *p* = 0.036). However, due to the limited sample size and age composition, predicting testicular vitality after testicular torsion using hematological parameters remains controversial; more prospective studies are needed to verify this.

This single‐center study has several limitations. First, ultrasound is highly dependent on the operator's experience. Therefore, we only evaluated the presence of blood flow in the patients' testes and did not assess the decrease in blood flow signal and normal blood flow in details. Second, surgery in patient was not performed by the same doctor, which may have led to selective bias in judging testicular retention during surgery. Finally, the postoperative follow‐up time might have been insufficient. Further follow‐up observations are needed for longer‐term changes in testicular volume and endocrine function.

In conclusion, this study determined the risk factors for testicular salvage after testicular torsion in our cohort. Increased symptom duration without intratesticular blood flow increased the monocyte count and spermatic cord torsion degree and decreased the success rate of testicular salvage. We constructed a nomogram based on the above factors and substituted the data of the verification cohort for ROC analysis. The AUC was 0.965 (95% CI 0.867–0.997), sensitivity was 90.5%, and specificity was 88.9%. This predictive model could provide parents and clinicians of children with testicular torsion with an exact probability based on the testicular salvage status of the child.

## AUTHOR CONTRIBUTIONS

**Pengyu Chen**: Conceptualization; Data curation; Formal analysis; Visualization; Writing—original draft; Writing—review & editing. **Weipeng Huang**: Formal analysis; Supervision; Validation; Writing—review & editing. **Yingying He**: Data curation; Investigation; Writing—review & editing. **Mengkui Sun**: Funding acquisition; Methodology; Writing—review & editing. **Xuerui Sun**: Investigation; Supervision; Writing—review & editing. **Yiyan Huang**: Validation; Writing—review & editing. **Shoulin Li**: Conceptualization; Funding acquisition; Methodology; Visualization; Writing—review & editing.

## FUNDING INFORMATION

This study was supported by the Shenzhen Fund for Guangdong Provincial High‐level Clinical Key specialties (grant number SZXK035); the National Natural Science Foundation of China (grant number U1904208); and Guangdong High‐level Hospital Construction Fund.

## CONFLICT OF INTEREST STATEMENT

None declared.

## APPROVAL OF THE RESEARCH PROTOCOL BY AN INSTITUTIONAL REVIEWER BOARD

Shenzhen Children's Hospital ethics committee (2022014).

## INFORMED CONSENT

Written informed consent was obtained from the guardians or parents before treatment.

## REGISTRY AND THE REGISTRATION NO. OF THE STUDY/TRIAL

N/A.

## ANIMAL STUDIES

N/A.

## References

[1] Zhao LC , Lautz TB , Meeks JJ , Maizels M . Pediatric testicular torsion epidemiology using a national database: incidence, risk of orchiectomy and possible measures toward improving the quality of care. J Urol. 2011;186:2009–2013. 21944120 10.1016/j.juro.2011.07.024

[2] Howe AS , Vasudevan V , Kongnyuy M , Rychik K , Thomas LA , Matuskova M , et al. Degree of twisting and duration of symptoms are prognostic factors of testis salvage during episodes of testicular torsion. Transl Androl Urol. 2017;6:1159–1166. 29354505 10.21037/tau.2017.09.10 PMC5760391

[3] Chen P , Huang W , Liu L , Chen N , Zhou G , Sun M , et al. Predictive value of hematological parameters in testicular salvage: a 12‐year retrospective review. Front Pediatr. 2022;10:989112. 36061382 10.3389/fped.2022.989112 PMC9428396

[4] Shunmugam M , Goldman RD . Testicular torsion in children. Can Fam Physician. 2021;67:669–671. 34521708 10.46747/cfp.6709669 PMC9683365

[5] Tian XM , Tan XH , Shi QL , Wen S , Lu P , Liu X , et al. Risk factors for testicular atrophy in children with testicular torsion following emergent orchiopexy. Front Pediatr. 2020;8:584796. 33262963 10.3389/fped.2020.584796 PMC7686235

[6] He M , Li M , Zhang W . Prognosis of testicular torsion orchiopexy. Andrologia. 2020;52:e13477. 31713875 10.1111/and.13477

[7] Arda IS , Ozyaylali I . Testicular tissue bleeding as an indicator of gonadal salvageability in testicular torsion surgery. BJU Int. 2001;87:89–92. 11121999 10.1046/j.1464-410x.2001.00021.x

[8] Lian BS , Ong CC , Chiang LW , Rai R , Nah SA . Factors predicting testicular atrophy after testicular salvage following torsion. Eur J Pediatr Surg. 2016;26:17–21. 26509312 10.1055/s-0035-1566096

[9] Niedzielski J , Balinska K , Wilk D , Slowikowska‐Hilczer J . The effect of the two‐stage laparoscopic Fowler‐Stevens operation on testicular growth and risk of atrophy in boys with intra‐abdominal testes. Arch Med Sci. 2022;18:666–671. 35591847 10.5114/aoms.2019.86596 PMC9102531

[10] Zvizdic Z , Aganovic A , Milisic E , Jonuzi A , Zvizdic D , Vranic S . Duration of symptoms is the only predictor of testicular salvage following testicular torsion in children: a case‐control study. Am J Emerg Med. 2021;41:197–200. 33221112 10.1016/j.ajem.2020.11.023

[11] Zheng WX , Hou GD , Zhang W , Wei D , Gao XL , Chen MH , et al. Establishment and internal validation of preoperative nomograms for predicting the possibility of testicular salvage in patients with testicular torsion. Asian J Androl. 2021;23:97–102. 32687070 10.4103/aja.aja_31_20 PMC7831831

[12] Goetz J , Roewe R , Doolittle J , Roth E , Groth T , Mesrobian HG , et al. A comparison of clinical outcomes of acute testicular torsion between prepubertal and postpubertal males. J Pediatr Urol. 2019;15:610–616. 31690483 10.1016/j.jpurol.2019.07.020

[13] Huang WY , Chen YF , Chang HC , Yang TK , Hsieh JT , Huang KH . The incidence rate and characteristics in patients with testicular torsion: a nationwide, population‐based study. Acta Paediatr. 2013;102:e363–e367. 23611668 10.1111/apa.12275

[14] Wang J , Deng Y , Gu N , Zhu H , Zhu X , Huang L , et al. Patient transfer influences the prognosis of pediatric patients operated for testicular torsion: a single‐center experience. Transl Pediatr. 2021;10:494–501. 33850808 10.21037/tp-20-287 PMC8039783

[15] Boettcher M , Krebs T , Bergholz R , Wenke K , Aronson D , Reinshagen K . Clinical and sonographic features predict testicular torsion in children: a prospective study. BJU Int. 2013;112:1201–1206. 23826981 10.1111/bju.12229

[16] Pinar U , Duquesne I , Lannes F , Bardet F , Kaulanjan K , Michiels C , et al. The use of doppler ultrasound for suspected testicular torsion: lessons learned from a 15‐year multicentre retrospective study of 2922 patients. Eur Urol Focus. 2022;8:105–111. 33663983 10.1016/j.euf.2021.02.011

[17] Dogra V , Bhatt S . Acute painful scrotum. Radiol Clin North Am. 2004;42:349–363. 15136021 10.1016/j.rcl.2003.12.002

[18] Preece J , Ching C , Yackey K , Jayanthi V , McLeod D , Alpert S , et al. Indicators and outcomes of transfer to tertiary pediatric hospitals for patients with testicular torsion. J Pediatr Urol. 2017;13:e1–e6. 10.1016/j.jpurol.2017.03.034 28527721

[19] Lin EP , Bhatt S , Rubens DJ , Dogra VS . Testicular torsion: twists and turns. Semin Ultrasound CT MR. 2007;28:317–328. 17874655 10.1053/j.sult.2007.05.008

[20] Castaneda‐Sanchez I , Tully B , Shipman M , Hoeft A , Hamby T , Palmer BW . Testicular torsion: a retrospective investigation of predictors of surgical outcomes and of remaining controversies. J Pediatr Urol. 2017;13:e1–e4. 10.1016/j.jpurol.2017.03.030 28476481

[21] He M , Zhang W , Sun N . Can haematologic parameters be used to predict testicular viability in testicular torsion? Andrologia. 2019;51:e13357. 31264256 10.1111/and.13357

[22] Jang JB , Ko YH , Choi JY , Song PH , Moon KH , Jung HC . Neutrophil‐lymphocyte ratio predicts organ salvage in testicular torsion with marginal diagnostic delay. World J Mens Health. 2019;37:99–104. 30584993 10.5534/wjmh.180049 PMC6305858

[23] Yilmaz M , Sahin Y , Hacibey I , Ozkuvanci U , Suzan S , Muslumanoglu AY . Should haematological inflammatory markers be included as an adjuvant in the differential diagnosis of acute scrotal pathologies? Andrologia. 2022;54:e14374. 35043470 10.1111/and.14374

